# Therapeutic outcomes of canal wall up mastoidectomy in combination with Type I tympanoplasty in otitis media

**DOI:** 10.12669/pjms.323.9780

**Published:** 2016

**Authors:** Liansheng Zhang

**Affiliations:** 1 Liansheng Zhang, Department of Otolaryngology, Zhumadian Center Hospital, Henan, 463000, China

**Keywords:** Canal wall up mastoidectomy, Hearing reconstruction, Otitis media, Tympanoplasty

## Abstract

**Objective:**

To evaluate the therapeutic effects in terms of disease clearance and hearing improvement of canal wall up mastoidectomy in combination with Type I tympanoplasty in otitis media.

**Methods:**

A total of 78 patients (81 ears) with otitis media were treated by canal wall up mastoidectomy in combination with Type I tympanoplasty. The postoperative tympanic membrane morphology, average of pure-tone hearing thresholds and average air-bone gap were used as the indices for evaluating therapeutic effects.

**Results:**

The patients were followed up for two years in average. All the tympanic membranes recovered, with the ear canals being dry. There were five cases (5 ears) of tympanic membrane retraction and one case of otitis media recurrence. Hearing was effectively recovered by 76.54% (62/81) after surgery.

**Conclusion:**

Combining canal wall up mastoidectomy with Type I tympanoplasty can treat otitis media safely and effectively due to high postoperative dry ear canal rate, satisfactory reconstruction of hearing and maintenance of ear morphology.

## INTRODUCTION

Mastoidectomy preserving the bony external auditory canal and tympanoplasty are referred to as closed technique or combined approach tympanoplasty that was first proposed by Sheehy and Janson,^1^,^2^ aiming to improve the classical mastoidectomy. The aim of this combined therapy is to eradicate infectious foci while retaining the bony external auditory canal. Compared with classical mastoidectomy, this method can avoid leaving the mastoid cavity open, without requiring frequent clearance of dry scabs in the surgical cavity. Meanwhile, hearing can be well maintained even patients take part in water sports or wearing hearing aid devices. Therefore, this method better meets the pathophysiological requirements. However, some otologists prefer open surgery to this protocol owing to high requirements of surgical technique and apparatus as well as inappropriate selection of indications that may lead to high recurrence rate.^3^,^4^

With the development of ear microsurgical techniques, infectious foci can be removed while preserving acoustic features-related structures and reconstructing hearing. After rational selection of indications, 78 patients (81 ears) with otitis media were treated by canal wall up mastoidectomy in combination with Type I tympanoplasty. They were then followed up to analyze the overall treatment outcomes

Our objective was to evaluate the therapeutic effects in terms of disease clearance and hearing improvement of canal wall up mastoidectomy in combination with Type I tympanoplasty in otitis media.

## METHODS

### Patient enrollment and Preoperative Examination

The surgical procedures were performed in accordance with the ethical standards of the responsible committee on human experimentation and with the Helsinki Declaration of 1975. Written consent was obtained from all patients. A total of 78 patients (81 ears) with otitis media who were enrolled in our hospital from March 2009 to January 2012 were treated by canal wall up mastoidectomy in combination with Type I tympanoplasty.

### Inclusion criteria

When otitis media, otoscopic examination through the external auditory canal showed the eardrum was inflammatory, manifested as redness, swelling and extrusion; reflection of the otoscopic light by the eardrum surface disappeared or attenuated; the eardrum had a white interface upon middle-ear hydrops; age older than 18 years.

### Exclusion criteria

Pregnant women; lactating women; patients complicated with severe cardiac, lung, liver or kidney diseases.

Before surgery, all patients were subjected to regular HRCT scan of the temporal bone, pure tone audiometry (or multi-frequency steady-state and bone-conduction auditory brainstem response) and Eustachian tube function (Valsalva method) examinations.

### Surgical Methods

**Fig.1:**
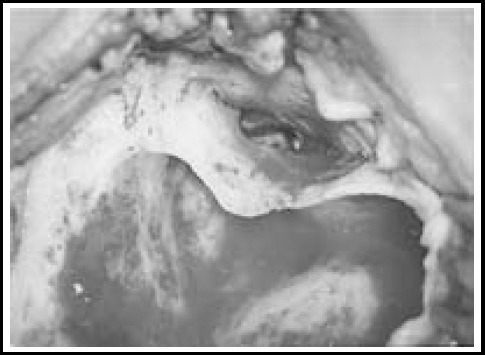
Lesions in the incudostapedial joint and tympanum exposed through the ear canal.

**Fig.2:**
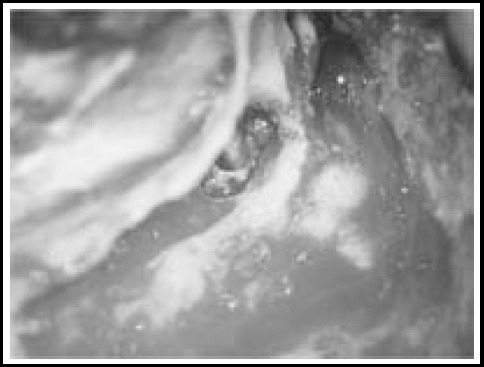
The incudomalleolar joint was exposed by opening the attic through the mastoid; lesions in the posterior tympanum and those surrounding the stapes were exposed by opening the facial recess.

**Fig.3:**
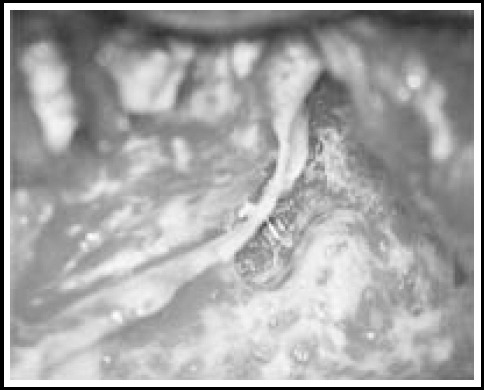
Posterior arch was worked up while retaining the posterior ear canal wall. Artificial ossicles were implanted after lesions were eliminated to reconstruct the tympanum.

Myoperiosteal flap on the muscle pedicle was prepared by using temporal fascia after making a postauricular incision. With the posterior ear canal wall preserved, the mastoid was contoured. Then the posterior ear canal wall was worn thin to gradually open the attic forward and to expose the incudomalleolar joint. Afterwards, tissues with pathological changes in the mastoid cavity and the attic were carefully removed. In the meantime, the posterior external ear canal wall was worn thin downward to expose the incudostapedial joint and stapes, and to eliminate the infectious foci in the posterior tympanum. Then the internal tympanum was exposed by lifting and pushing the tympanic flap of ear canal skin through the external auditory canal, after which lesions were cleared from two sides of the tympanum and mastoid (Fig.1 and Fig.2). The ossicles should be removed simultaneously when involved. After the malleus was cut off, lesions of the anterior malleus recesses were removed. In case of severe tympanic lesions, the posterior arch should be worked up for complete exposure. The cavity receiving surgery was rinsed repeatedly after the lesions were eliminated. According to the loss outcomes of ossicles, the ossicular chain was reconstructed and artificial ossicles were fixed by antibiotic gelatin sponges (Fig.3). Thereafter the tympanic membrane was repaired by inserting a fascia into the ear canal, and the mastoid cavity was sutured by pulling the myoperiosteal flap backward before a thin silicone tube was inserted for drainage. In the meantime, the ear canal was packed with gelatin sponges and ear swellable sponges at proper sizes. Particularly, the patients suffering from erosion of the ear canal wall and those with widely distributed infectious foci that could not be completely cleared due to preservation of the posterior external ear canal wall were excluded. Such patients were then subjected to open surgeries to grind off the posterior ear canal wall.

### Observation of Clinical Therapeutic Effects

The tympanic membrane was assessed postoperatively according to the following criteria. 1) Cured: The tympanic membrane did not congest after surgery, and purulent secretions in the external ear canal and otitis media symptoms disappeared; 2) remitted: the tympanic membrane still mildly congested after surgery, but purulent secretions in the external ear canal and otitis media symptoms were significantly mitigated; 3) ineffective: the tympanic membrane was still congested after surgery, and purulent secretions or clinical symptoms were unalleviated or even aggravated.

All patients were followed up, i.e. otoscopic examination in the postoperative 1st and 3rd months as well as pure tone audiometry in the postoperative 6th month. One year later, pure tone audiometry was performed once every six months. The outcomes of lesion removal, preservation of posterior ear canal wall structures, tympanic membrane and hearing were recorded. Averages of air-conduction thresholds and average air-bone gaps at 0.5, 1, 2 and 4 kHz detected in the last follow-up were used to evaluate the postoperative hearing. In addition, the hearing changes were observed by comparing the values with the pure-tone hearing thresholds before surgery.

## RESULTS

There were 42 males and 36 females between 13-56 years of age [average: (35.6±7.3)]. Disease course: 5 months ~ 30 years (Table-I). Lesions of all the patients were successfully eliminated and the anatomical structures of the posterior external ear canal wall were retained, without any perioperative complications. During two years of follow-up, all the tympanic membranes were recovered, with the ear canals being dry. Morphology of the ear canal was kept normal after epithelization. Five patients (5 ears) who suffered from tympanic membrane retraction were treated by improving the Eustachian tube functions. One case had recurrent otitis media 9 months after the surgery, who could not be treated well by conservative protocols. CT examination disclosed shadows of the middle ear and mastoid soft tissues. Therefore, radical open surgery was conducted subsequently. All patients had dry ear canals within postoperative 6.8 weeks in average.

**Table-I:** Patients data.

*Patient*	*Gender*	*Average age*	*Number of ears*	*Average disease course (year)*
42	Male	37.1±3.3	43	21.1±7.3
36	Female	35.7±5.3	38	23.3±1.5

Ossicular chains in 48 ears were preserved, partial ossicular replacement prostheses were placed in 31 ears, and total ossicular replacement prostheses were placed in two ears. There were various inflammatory lesions (e.g. granulation tissues, cholesterol granuloma, tympanosclerotic focus and adhesive tissues) in 51 ears and restricted cholesteatoma in 30 ears. Hearing was rendered as effective increase when air-bone gap was decreased by 15 dB after surgery. The preoperative and postoperative pure-tone hearing thresholds are summarized in Table-II. The average air-conduction thresholds and air-bone gaps significantly reduced after surgery, and the latters of 62 patients decreased by ≥15 dB. In other words, the rate of effective hearing increase was 76.54% (62/81).

**Table-II:** Hearing examination results.

*Group (n)*	*Before*		*After*	

	*Air-conduction threshold*	*Air-bone gap*	*Air-conduction threshold*	*Air-bone gap*
Various inflammatory foci(51)	35.37±9.54	34.38±10.02	21.48±8.02	17.57±6.46
Restricted cholesteatoma(30)	33.82±11.06	29.74±10.48	20.36±8.34	15.73±7.34

## DISCUSSION

Otologists have endeavored to better treat otitis media, a common otological disease. Canal wall up mastoidectomy, which modifies the classical method, is devoted to preservation or improvement of the physiological and auditory functions besides elimination of infectious foci. This protocol, although capable of both radical treatment and functional reconstruction, requires accurate selection of indications, adeptness in anatomy and surgical operations, as well as sophisticated apparatus. In this study, the long-term effects of this protocol were evaluated by following up eligible patients who were free from lesions with reconstructed hearing for two years in average. In addition to complete removal of infectious foci in all the selected patients (ears), the normal anatomical structures of the posterior external ear canal wall were preserved and the rate of effective hearing increase reached 76.54% (62/81), similar to the results of a previous study.^5^

Similarly, it has been reported that the recurrence rates of canal wall up mastoidectomy ranged between 5%-40%,^6^-^8^ mostly in anterior attic recesses and facial nerve recesses. Therefore, it is of great significance to sufficiently open the facial recess, and to completely expose the concealed lesions by removing the posterior arch and the malleus if necessary. During the surgery, essential structures such as facial nerves were not injured owing to well-trained, proficient operations. In this study, during the two year follow-up, only one patient with poor Eustachian tube function succumbed to otitis media recurrence because of persistent postoperative tympanic negative pressure. The low recurrence rate can mainly be attributed to the radical treatment.^8^

Moreover, indications were rationally selected based on temporal bone CT and hearing examinations together with clinical characteristics and intraoperative changes. Canal wall up mastoidectomy in combination with tympanoplasty is applicable to the treatments of all kinds of chronic otitis media in the case of intact external auditory canal bone wall, Eustachian tube patency and relatively restricted lesions. However, open surgery and ossicular chain reconstruction should be conducted instead for middle ear-involved external auditory canal cholesteatoma, widely invasive cholesteatoma and infectious foci, intracranial and extracranial complications, and necrotic osteitis, aiming to allow dry ear canal by eliminating foci.^9^ Particularly, surgeons should refrain from canal wall up mastoidectomy for the patients complicated with persistent Eustachian tube functional disorders.^10^

Meanwhile, elimination of lesions should be given first priority over surgeries that can retain the original ear anatomical structures. More importantly, even if open surgery in combination with ossicular chain reconstruction can basically be comparable to canal wall up mastoidectomy due to the application of artificial ossicles and the advance in surgical techniques,^11^ with low risks of recurrence also. Hence, mastoids should be opened timely if lesion tissues cannot be entirely removed with the posterior ear canal wall preserved.

The novel protocol is generally performed within narrow cavities under a small visual field, which thus easily causes complications in the absence of sufficient knowledge and skilled surgeons. The complications commonly include facial nerve injury, damage of the labyrinthine segment, cerebrospinal fluid leakage, cholesteatoma recurrence and middle ear adhesions, etc.^12^ Therefore, it is of great significance to circumvent complications by being more adept at surgical skills. Canal wall up mastoidectomy is mainly restricted in the limited exposure of key sites receiving treatment, high requirements of surgical skills, time-consuming procedure, and strong possibility of incomplete lesion removal. Given the high recurrence rate, particular attention should be paid to selection of indications and surgical approaches.

## CONCLUSION

When indications are rationally selected, retaining the bony external auditory canal by skilled surgeons is more conducive to preservation of ear morphology and reconstruction of normal tympanic and acoustic structures compared with traditional radical treatment protocols.

## References

[1] Sheehy J, Patterson ME (1967). Intact canal wall tympanoplasty with mastoidectomy. Larygoscope.

[2] Jansen C (1968). The combined approach for tympanoplasty. J Laryngol Otol.

[3] Bennett M, Warren F, Haynes D (2006). Indications and technique in mastoidectomy. Otolaryngol Clin North Am.

[4] Ayache S, Tramier B, Strunski V (2008). Otoendoscopy in cholesteatoma surgery of the middle ear:what benefits can be expected?. Otol Neurotol.

[5] Darrouzet V, Duclos JY, Portmann D, Bebear JP (2000). Preference of closed technique in the management of cholesteatoma of the middle ear in children:a retrospective study of 215 consecutive patients treated over 10 years. Am J Otol.

[6] Naclerio R, Neely JG, Alford BR (1981). A retrospective analysis of the intact canal wall tympanoplasty with mastoidectomy. Am J Otol.

[7] Wilson KF, Hoggan RN, Shelton C (2013). Tympanoplasty with intact canal wall mastoidectomy for cholesteatoma:long-term surgical outcomes. Otolaryngol Head Neck Surg.

[8] Cao Y, Shao C, Song Y, Bai C, He L (2015). Clinical analysis of patients with primary ciliary dyskinesia in mainland China. Clin Respir J.

[9] Stew BT, Fishpool SJ, Clarke JD, Johnson PM (2013). Can early second-look tympanoplasty reduce the rate of conversion to modified radical mastoidectomy?. Acta Otolaryngol.

[10] Parmekar S, Hazarika P, Viswanathan A, Balakrishnan R (1997). Mastoid obliteration using temporo-parietal fascia flap-our experience. Indian J Otolaryngol Head Neck Surg.

[11] Heo KW, Kang MK, Park JY (2014). Alternative to canal wall-down mastoidectomy for sclerotic mastoid cavities:epitympanoplasty with mastoid obliteration. Ann Otol Rhinol Laryngol.

[12] Kim MB, Choi J, Lee JK (2010). Hearing outcomes according to the types of mastoidectomy:a comparison between canal wall up and canal wall down mastoidectomy. Clin Exp Otorhinolaryngol.

[13] Hinohira Y, Yanahigara N, Gyo K (2007). Improvements to staged canal wall up tympanoplasty for middle ear cholesteatoma. Otolaryngol Head Neck Surg.

